# Suppression of neuroinflammation by an allosteric agonist and positive allosteric modulator of the α7 nicotinic acetylcholine receptor GAT107

**DOI:** 10.1186/s12974-021-02149-4

**Published:** 2021-04-26

**Authors:** Tehila Mizrachi, Oshrit Marsha, Karen Brusin, Yael Ben-David, Ganesh A. Thakur, Adi Vaknin-Dembinsky, Millet Treinin, Talma Brenner

**Affiliations:** 1 Department of Neurology, The Agnes Ginges Center for Human Neurogenetics, Hadassah University Hospital and Hebrew University Medical School, Jerusalem, Israel; 2 Department of Medical Neurobiology, Hebrew University—Hadassah Medical School, Jerusalem, Israel; 3 Pharmaceutical Science, Bouve College of Health Science, Northeastern University, Boston, USA

**Keywords:** α7 Nicotinic acetylcholine receptor, Selective allosteric agonist for α7 nAChR, Multiple sclerosis, B cells, Central nervous system inflammation, Immune cholinergic system

## Abstract

**Background:**

The α7 nicotinic acetylcholine receptor (α7 nAChR) negatively regulates the synthesis and release of pro-inflammatory cytokines by immune cells. Our previous studies showed that in encephalitogenic T cells, α7 nAChR expression is upregulated and that activation of the cholinergic system can attenuate experimental autoimmune encephalomyelitis (EAE). GAT107 is an allosteric agonist and positive allosteric modulator (ago-PAM) of α7 nAChR that can produce persistent activation of this receptor. Therefore, in the present study, we investigated the effect of GAT107 on neuroinflammation in EAE, the animal model used for the study of multiple sclerosis (MS) via α7 nAChR, and the inflammatory pathways involved.

**Methods:**

EAE was induced by administration of myelin oligodendrocyte glycoprotein (MOG_35–55_) in C57BL/6 mice. EAE mice were treated with the ago-PAM GAT107 or a placebo for 9 days, starting from the day of EAE induction. Clinical assessment and immunological evaluation of immune cells and cytokine production was performed.

**Results:**

Following activation of the α7 nAChR by GAT107 during EAE, disease severity was significantly reduced by 70% and was correlated with a reduction in the extent of neuroinflammation in the CNS. The treatment reduced encephalitogenic T cell proliferation and the production of pro-inflammatory cytokines, as well as increased the production of the anti-inflammatory cytokine IL-10. Furthermore, the expression of immune cell markers was altered by GAT107 treatment, which induced a significant reduction in macrophages, dendritic cells, and B cells, as well as a reduction in anti-MOG_35–55_ antibodies. Additionally, GAT107 was found to directly activate α7 nAChR in murine macrophage RAW264.7 cells and in human PBMCs derived from MS patients and healthy donors.

**Conclusions:**

Our results show that GAT107 can be a useful molecule for harnessing the cholinergic anti-inflammatory pathway for long-lasting and wide-ranging modulation and downregulation of neuroinflammation in EAE.

## Background

Multiple sclerosis (MS) is a classical inflammatory demyelinating disease of the central nervous system (CNS). Experimental autoimmune encephalomyelitis (EAE) is a complex condition that mimics the key pathological features of MS, which is characterized by interactions between a variety of immunopathological and neuropathological mechanisms, such as inflammation, demyelination, axonal loss, and gliosis. This model has been helpful for the development of new therapies for MS [1–4] and will also be applied in the present study.

Our previous work used the EAE model to demonstrate the novel anti-inflammatory effects of the non-neuronal cholinergic system in EAE and neuroinflammation [5–7]. The cholinergic system modulates interactions between the CNS and the immune system and plays a role in the modulation of various immune functions. This forms the basis of nerve–immune interactions, whereby the nervous system plays a part in controlling the magnitude and quality of immune responses. In our previous research, cholinesterase inhibitors (ChEIs) and nicotine [5, 6] were shown to attenuate the clinical symptoms of EAE and to inhibit T cell reactivity and cytokine production. These cholinergic effects were dependent on the activation of α7 nicotinic acetylcholine receptors (nAChRs) on immune cells. Initially, α7 nAChR was identified as an anti-inflammatory target in macrophages [8, 9]; later, it was shown to be expressed and function in various immune cells, such as T cells [5], dendritic cells, and B cells [9]. Indeed, it has been demonstrated that nicotinic receptors in the periphery inhibit auto-reactive T cell proliferation and alter their cytokine profile [10]. In the CNS, nicotine exposure reduces the number of dendritic cells, infiltrating monocytes, and resident microglial cells, and downregulates the expression of MHC class II molecules [11]. Additionally, with regard to EAE, Godin et al. reported that the non-competitive nAChR antagonist mecamylamine and the silent agonist 1-ethyl-4-(3-(bromo)phenyl)piperazine (m-bromo PEP) reduced pro-inflammatory responses and ameliorated EAE [12]. All these findings indicate that therapeutic agents that target nAChRs may have potential for the treatment of neuroinflammatory diseases such as MS.

α7 nAChR has unique physiological and pharmacological properties including high permeability for calcium and sodium, and rapid and reversible desensitization [13]. In addition, α7 nAChRs are activated by acetylcholine (ACh) and selectively by choline, which is a precursor and breakdown product of ACh. New approaches for the therapeutic targeting and activation of α7 nAChRs have identified several α7-selective positive allosteric modulators (PAMs) that overcome the limitations of ligands that directly activate α7 nAChRs [14]. For example, GAT107 that was used in the current study, is a dual allosteric agonist and a positive allosteric modulator (ago-PAM) of α7 nAChR [15]. Activation of α7 nAChRs by GAT107 stimulates the anti-inflammatory pathway of the immune cholinergic system and can modulate immune responses [16], and recently, Quadri et al. also showed that GAT107 directly caused opening of α7 nAChR channel [17]. This unique agonist was also shown to have long-lasting effects on receptor activity [16].

The cascade of events leading to CNS inflammatory lesions is initiated by CD4+T cells that are activated in the periphery towards myelin antigens. These encephalitogenic T cells then migrate to the CNS, and upon additional activation with resident antigen-presenting cells (APC) such as microglia, astrocytes, and a subpopulation of dendritic cell, induce plaque formation [18]. The α7 nAChR is expressed by naïve T cells, as well as by macrophages. Its expression is augmented following immune activation. Nicotine and ACh could inhibit T cell proliferation in response to mitogen, by activation of α7 nAChR [19]. The α7 nAChR affect different immune cells including glial cells, which can participate in immune reaction. The various effects of the α7 nAChR on the different cell types are summarized in the review [9].

B cells, which are best known for their capacity to produce antibodies, also infiltrate MS lesions in the CNS. Some B cells produce antibodies against myelin antigens and, thus, can lead to demyelination and axonal damage [20]. Besides antibody production, the functions of B cells in MS include antigen presentation, co-stimulation, and production of pro-inflammatory cytokines, such as IL-6, or anti-inflammatory cytokines such as IL-10 [21, 22].

In EAE, B cells have distinct pathogenic or regulatory functions that are dependent on the stage of the disease [23]. This is supported by findings in B cell-deficient mice, which exhibit an exacerbated disease course that is probably a result of the disruption of the regulatory functions of B cells and their capacity to produce IL-10 [20, 24, 25]. However, B cell depletion after EAE induction ameliorates disease severity due to the pathogenic role of memory B cells, which facilitate T cell reactivation during later stages of the disease [26–29]. Recently, there is increasing interest in the significant role of B cells in MS; therefore, we tested in the present study how the ago-PAM GAT107 influences B cell populations and their functions in EAE.

In the present study, we investigated the effects of the ago-PAM GAT107 on the cholinergic immune system in EAE mice. Our findings indicate that treatment with this specific agonist had beneficial effects on the clinical severity of EAE, inflammatory processes in the CNS, and the pattern of immune cell sub-populations. In addition to its immunomodulatory effects on the MS model animals, GAT107 demonstrated a direct effect on the cellular inflammatory responses of macrophages and human peripheral blood mononuclear cells (PBMCs). Therefore, our findings indicate that GAT107 has therapeutic potential in the treatment of inflammatory diseases of the CNS through activation of the cholinergic immune system.

## Methods

### Mice

Female C57BL/6 mice (7–8 weeks old) were purchased from Harlan Laboratories, Rehovot, Israel, and housed under specific pathogen-free conditions in the animal facility of the Hebrew University Medical School, Jerusalem, Israel, in accordance with the National Institutes of Health guidelines for the care and use of laboratory animals.

### EAE induction and clinical evaluation

EAE was induced in 8-week-old female C57BL/6 mice by subcutaneous administration of 250 μg of myelin oligodendrocyte glycoprotein (MOG_35–55_) (synthesized by Sigma Laboratories, Israel) emulsified in complete Freund’s adjuvant (CFA) containing 5 mg/ml heat-killed *Mycobacterium tuberculosis*. The MOG_35–55_ peptide was administered in the left paralumbar region. Immediately after the first injection and 48 h later, 200 ng of the pertussis toxin (List Biological Industries, San Diego, CA, USA) was intraperitoneally (i.p.) administered as previously described [3]. All the animals were examined daily and evaluated for clinical signs of EAE. The clinical status of the mice was graded as follows: 0 = no clinical disease, 1 = tail weakness, 2 = hind limb weakness sufficient to impair righting, 3 = single-limb plegia, 4 = paraplegia with forelimb weakness, 5 = quadriplegia, and 6 = death. According to the ethical requirements, mice that reached stage 4 were euthanized.

The clinical evaluation of the EAE mice was performed by a non-informed investigator; the division of the mice into the groups of the placebo or GAT 107 treatment was done in the same manner.

### GAT107 treatment

GAT107 was dissolved in a mixture of ethanol, Emulphor-620 (Rhone-Poulenc Inc., Princeton, NJ, USA), and distilled water at a vol:vol:vol ratio of 1:1:18 [16]. GAT107 or placebo were intraperitoneally administered twice a day at a dose of 10 mg/kg [16], starting from the EAE induction day, for 8 consecutive days. Mice were followed clinically as described in the previous subsection.

### Evaluation of CNS pathology

On the day of sacrifice, lumbar spinal cords, where inflammatory foci predominate in our model, were harvested, fixed in 4% paraformaldehyde, dehydrated, and embedded in paraffin. Longitudinal sections containing both gray and white matter, which cover the majority of the length of the spinal cord, were stained with hematoxylin-eosin as previously described [30]. Slides were evaluated under a light microscope (Axioplan-2; Zeiss), and inflammatory foci containing at least 20 perivascular mononuclear cells were counted in each section.

### Mouse lymphocyte proliferation assay

Pooled lymph node cells (LNCs) were prepared from the inguinal, axillary, and mesenteric lymph nodes or from spleens derived from naive mice or from mice that had been subcutaneously inoculated 9 days earlier with the MOG_35–55_ peptide in CFA. The in vitro lymphocyte response was assayed in triplicate wells of 96-well flat-bottom microtiter plates as previously described [31]. A total of 2 × 10^5^ LNCs, suspended in 0.2 ml RPMI supplemented with 5% fetal calf serum (FCS), were added to each well with or without 100 μg/ml of the MOG_35–55_ peptide. At 48 h after seeding, 1 μCi [H^3^] thymidine (Amersham Pharmacia Biotech, Amersham, Buckinghamshire, UK) was added to each well as a radioactive marker, and the plates were incubated for an additional 18 h. The plates were then harvested with a semi-automated harvester onto a glass fiber filter, and radioactivity was assessed with a liquid scintillation assay.

### Cytokine secretion assay

The culture media containing splenocytes or LNCs were incubated in the presence of MOG_35–55_, anti-CD3 antibody, ConcavalinA (ConA), or lipopolysaccharide (LPS). Supernatant was collected at different time points: at 24 h for the IFNγ and IL-17 assay, and at 72 h for the IL-10 assay. The cytokine concentrations were determined with ELISA kits for each of the cytokines (Biolegend, San Diego, CA, USA).

### Flow cytometry analysis

For leukocyte surface marker determination, pooled LNCs or splenocytes were obtained from mice (as described in the mouse lymphocyte proliferation assay). Cell suspensions were prepared as described previously [31]. For immune phenotyping, the following antibodies were used: anti-CD4 FITC, anti-CD8 FITC, anti-CCR5 PE, anti-B220 FITC, anti-CD11c PE, anti-MHC class II FITC, and anti-CD11b PE (all from eBioscience, San Diego, CA, USA). Stained cells were counted with a FACS Calibur flow cytometry kit (FC500, Beckman Coulter).

### Quantitative real-time PCR

Total RNA was prepared using a GeneJET RNA purification kit (Thermo Fisher Scientific), and cDNA was prepared from 1 μg of total RNA with a qScript^TM^ cDNA synthesis kit (Quanta Bio). The PCR reaction mixture contained 50 ng cDNA, 300 nM of the appropriate forward and reverse primers (Sigmae Genosys Ltd., Cambridgeshire, UK), and 6 μl of the master mix buffer containing nucleotides, Taq polymerase, and SYBR Green (SYBR Green fast mix ROX, Quanta bio) in a total volume of 10 μl. Gene amplification was carried out using the StepOnePlus™ real-time PCR system (Thermo Fisher Scientific). The amplification protocol was as follows: 10 min at 95 °C followed by 40 cycles of a two-step loop (20 s at 95 °C and 1 min at 60 °C). The results are expressed as relative quantification (RQ) values, that is, as the fold increase in gene expression in samples from MOG_35–55_-treated cells compared to the gene expression in control cells. The results for gene expression were normalized to the HPRT gene, as its expression did not change under the experimental conditions.

The following primers were used:

1. HPRT
  Forward: 5′-TCCTCCTCAGACCGCTTTT-3′
  Reverse: 5′-CCTGGTTCATCATCGCTAATC-3′
2. GATA-3
  Forward: 5′-GCAGAAAGCAAAATGTTTGCTTC-3′
  Reverse: 5′-GAGTCTGAATGGCTTATTCACAAATG-3′
3. RORγt
  Forward: 5′-CCCTGGTTCTCATCAATGC-3′
  Reverse: 5′-TCCAAATTGTATTGCAGATGTTC-3′
4. T-bet
  Forward: 5′-CAGTTCATTGCAGTGACTGCCTAC-3′
  Reverse: 5′-CAAAGTTCTCCCGGAATCCTTT-3′
5. FoxP3
  Forward: 5′-AGAAGCTGGGAGCTAT-3′
  Reverse: 5′-GCTACGCTGCAGCAAG-3′
6. TGF-β
  Forward: 5′-TCAGACATTCGGGAAGCAGT-3′
  Reverse: 5′-ACGCCAGGAATTGTTGCTAT-3′
7. BAFF
  Forward: 5′-TGCTACTCGGCTGGCATCGC-3′
  Reverse: 5′-GCGCCGGCTCCGTTTCTCAT-3′
8. BAFF-R
  Forward: 5′-GACCCTGGGTCTAGTGA-3′
  Reverse: 5′-GTAGGAGCTGAGGCATGAGG-3′
9. BCMA
  Forward: 5′-ATCTTCTTGGGGCTGACCTT-3′
  Reverse: 5′-CTTTGAGGCTGGTCCTTCAG-3′
10. APRIL
  Forward: 5′-CTGGAGGCCAGGGAGACAT-3′
  Reverse: 5′-GCACGGTCAGGATCAGAAGG-3′
11. TACI
  Forward: 5′-CTACTACACACTGGGGGTCT-3′
  Reverse: 5′-CTCCTGAGTGGGAGAACTGC-3′
12. OPN
  Forward: 5′-AGCAAGAAACTCTTCCAAGCAA-3′
  Reverse: 5′-GTGAGATTCGTCAGATTCATCCG-3′

### ELISA for serum anti-MOG_35–55_ antibody

The level of MOG-specific antibody during the course of EAE was measured using specific ELISA as previously described [3]. Individual wells in 96-well flat-bottom ELISA microplates (Nunc, Denmark) were coated with 100 μl of 1 μg/ml MOG_35–55_ in 0.17 M borate coating buffer (pH 8) and incubated overnight at 4 °C. The plates were then blocked for 90 min at room temperature (RT) with phosphate-buffered saline (PBS) containing 1% (w/v) bovine serum albumin (BSA). Next, the plates were washed with saline containing 0.05% Tween 20 and PBS, and 100 μl of serum diluted to 1:200 or 1:100 in dilution buffer (PBS containing 1% BSA and 0.05% Tween 20) was added to the plates. Serum from naive mice served as the negative control. The plates containing the serum solutions were then incubated overnight at 4 °C and washed (with saline containing 0.05% Tween 20 and PBS). Following this, they were incubated for 90 min at RT with 100 μl of alkaline phosphatase-conjugated goat anti-mouse IgG (Fc specific) or anti-IgM (μ chain specific) (Sigma, USA), diluted 1:35,000 in dilution buffer. The reaction product was visualized using *p*-nitrophenyl phosphate (1 mg/ml; Sigma, USA) in 1 M diethanolamine buffer (pH 9.8) containing 0.5 mM MgCl_2_ and incubated for 50 min at RT. The reaction was terminated by the addition of 30 μl of 3 N NaOH. Absorbance at 405 nm was measured in a micro-ELISA reader.

### Direct effect of GAT107 on the inflammatory responses of murine macrophage RAW cells

RAW 264.7 cells, a line of murine macrophage adherent cells, were grown in complete culture media consisting of RPMI 1650 supplemented with 10% fetal bovine serum, 1% glutamine, and 1% PenStrep. All experiments with the cells were conducted in a sterile laminar hood. On the first day of each experiment, 1.5 × 10^6^ cells were plated in 6-well dishes containing 3 ml of complete culture media, as previously described [32]. The following day, the medium was changed, and ACh (100 μM), GAT107 (10 μM), and the nicotinic antagonist methyllycaconitine (MLA) (10 nM) were added to the wells and incubated at 37 °C in a 5% CO_2_ atmosphere for 0.5 h. Subsequently, LPS (25 μg/ml) was added to the wells and incubated for an additional 9 h under the same conditions. Supernatants were collected at the end of incubation and frozen at − 80 °C until the IL-6 concentration was determined with the ELISA kit (Biolegend, San Diego, CA, USA).

### Effects of GAT107 and ACh on human PBMCs

Peripheral blood samples from 24 healthy donors and 32 MS patients were collected in EDTA tubes (Vacuette; Greiner Bio-one). PBMCs were isolated by Ficoll–Hypaque density gradient centrifugation and re-suspended in complete culture media consisting of RPMI 1640 medium supplemented with 5% fetal calf serum(FCS), penicillin + streptomycin (100 i.u./ml), MEM non-essential amino acids (1:100), pyruvate (1 mM), l-glutamine solution (2 mM), and β-mercaptho-ethanol (3 nM). The isolated PBMCs (1 × 10^6^) were immediately placed in 24-well plates in a volume of 0.5 ml of complete culture medium. ACh (100 μM) and GAT107 (10 μM) were added to the wells, and the plates were incubated at 37 °C in a 5% CO_2_ atmosphere for 0.5 h. Subsequently, ConA (5 μg/ml) was added to the wells and the plate was incubated for an additional 24 h in the same conditions. Supernatants were collected at the end of incubation and frozen at − 80 °C until assay. The IL-17 and IL-6 concentrations were determined with the ELISA kit (Biolegend, San Diego, CA, USA). The HD and MS characteristics are presented in Table 1.

The present study was approved by the local Helsinki Committee and the Israeli Institutional Review Board (approval number HMO0298-17).

### Statistical analysis

The data were analyzed using Student’s *t*-test and one-way ANOVA, according to Dunnett, and the Fisher exact test. *p* < 0.05 was considered to indicate statistical significance.

## Results

### Amelioration of EAE with GAT107 treatment

**Fig. 1:**
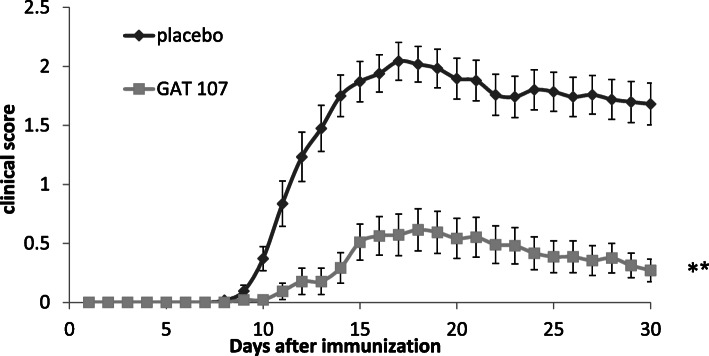
Amelioration of the clinical course of EAE on treatment with the ago-PAM GAT107. EAE was induced in wild-type (WT) mice by immunization with myelin oligodendrocyte glycoprotein (MOG)_35–55_. The animals were given either placebo or GAT107 (10 mg/kg) twice daily by intraperitoneal injection. The results are expressed as the mean clinical score ± standard error (SE) of data from four separate experiments (*n* = 29 in the placebo group and *n* = 24 in GAT107-treated group) (**p < 0.001)

To assess the influence of α7 nAChR activation during neuroinflammation in EAE, we used the ago-PAM GAT107. EAE model mice were divided into two groups: one group was treated with placebo, while the second group was treated with GAT107. The treatment consisted of a total of 16 intraperitoneal injections, starting from the day of EAE induction, for 8 days, twice a day, at a dose of 10 mg/kg [16]. As shown in Fig. 1 and Table 2, treatment with GAT107 remarkably reduced mean disease severity (which was the average daily clinical score) by 78% compared with the placebo group (*p* < 0.001). Similarly, the cumulative score, which was calculated as the number of days that the animal was sick multiplied by the clinical score, was reduced by 76% with GAT107 treatment (*p* < 0.001). Additionally, the max score, which indicated the average maximum clinical score for each mouse, was reduced by 70% (*p* < 0.001), and the day of onset (the average of the first day of clinical symptom appearance) was delayed by 3 days in the GAT107-treated group (*p* < 0.05) (Table 2). These effects persisted long after GAT107 administration was discontinued.

**Table 1:** Epidemiological and clinical characteristic of the MS patients and the healthy donors

	Healthy donors	MS patients
***n***	24	32
**Female/male**	19/5	23/9
**Age (mean ± SE)**	36.4 ± 3.2	40.1 ± 2.5
**Disease duration (mean ± SE)**	--	7.4 ± 1.1
**EDSS (mean ± SE)**	--	2.2 ± 0.3

**Table 2:** Influence of treatment with GAT107 on the clinical parameters of EAE

	Mean severity	Cumulative score	Max score	Day of onset
Placebo	1.8 ± 0.15	33.8 ± 2.9	2.25 ± 0.14	11.2 ± 0.5
GAT107	0.4 ± 0.12**	8.1 ± 2.4**	0.68 ± 0.18**	14.2 ± 0.6*

*p < 0.05, **p < 0.001

Mice were intraperitoneally administered GAT107 or saline (control group) at a dose of 10 mg/kg twice a day for 8 consecutive days. Mean severity is the average of the daily clinical score of each group. The cumulative score is calculated as the number of days that the animal was sick multiplied by the clinical score. The max score indicates the average maximum clinical score for each mouse in each group. The day of onset indicates an average for the first day of clinical symptoms appearance of each group (*p < 0.05, **p < 0.001)

### Inhibition of CNS inflammation by GAT107

**Fig. 2:**
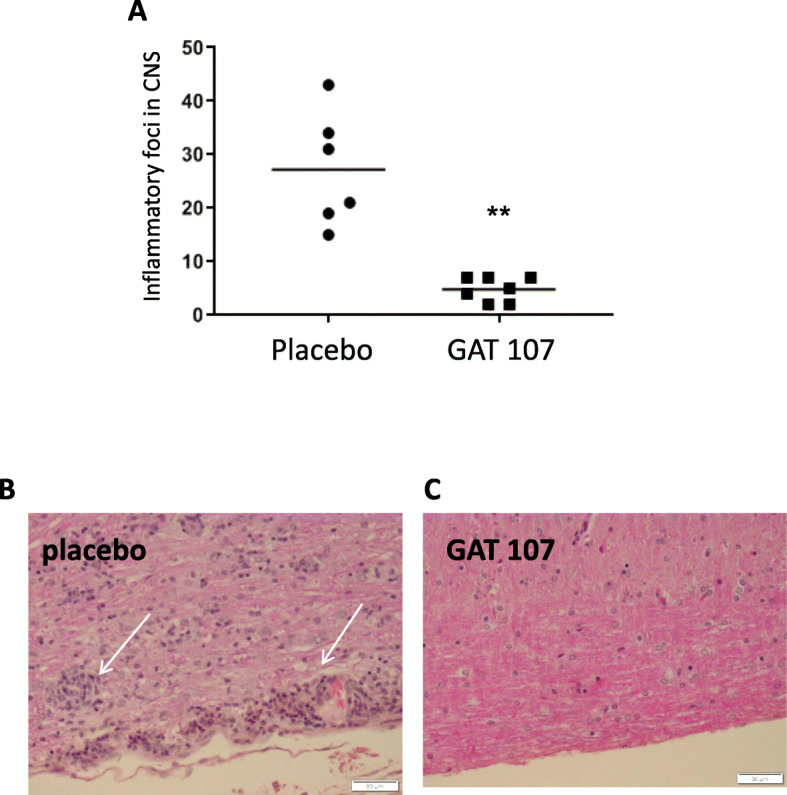
Neuropathological features of spinal cords in EAE mice treated with GAT107. Spinal cords of mice from the placebo- and GAT107-treated groups were removed at day 30 after EAE induction. **a** Treatment with GAT107 (10 mg/day) for 8 days improved CNS inflammation and reduced the number of EAE lesions. Representative images (with hematoxylin and eosin staining) at × 20 magnification are shown for the **b** placebo- and **c** GAT107-treated cells. The arrows indicate the foci (*n* = 6 in the placebo group and *n* = 7 in GAT107-treated group) (**p < 0.001)

The significant difference in clinical disease score between the GAT107-treated and placebo-treated mice was confirmed by the findings from histological analysis of spinal cord tissue resected at day 30 of disease induction. In the placebo-treated group, pronounced perivascular, and meningeal infiltration was observed (Fig. 2), whereas the extent of inflammation was much lesser in the GAT107-treated group. Indeed, GAT107 treatment resulted in an 80% decrease in the number of inflammatory foci compared with the placebo-treated group (*p* < 0.001) (Fig. 2).

### Inhibitory effect of GAT107 on MOG-specific T cell proliferation and pro-inflammatory cytokine production

**Fig. 3:**
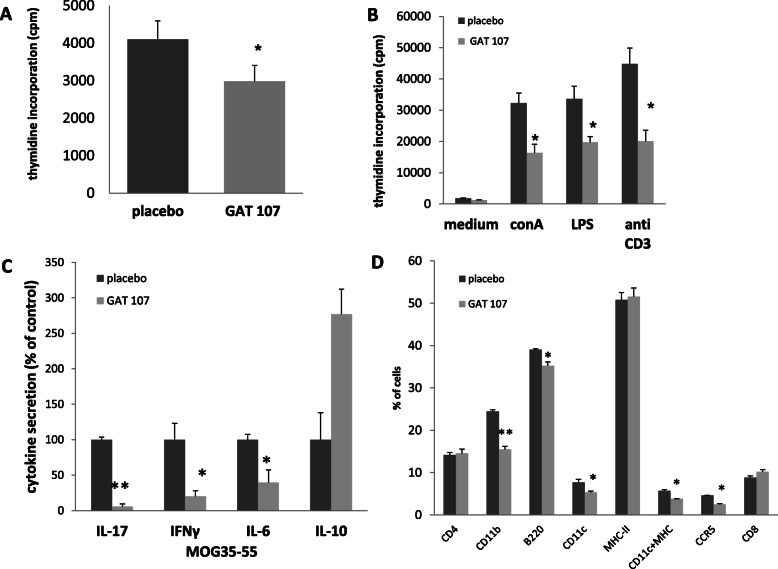
Reduction in T cell ex vivo reactivity following GAT107 treatment. Proliferation of lymphocytes from placebo- and GAT107-treated mice was assessed based on [3H] thymidine incorporation in the presence of MOG_35–55_ (**a**) or in the presence of ConA, anti-CD3 antibody, or LPS (**b**). IL-17, IFN-γ, IL-6, and IL-10 levels following MOG_35–55_ stimulation were measured by ELISA, and the results are expressed as percentage of the mean secretion ± SE value of the placebo-treated group based on data from three separate experiments (**c**) (*n* = 6 for each group). The expression of immune cell surface markers was assessed by FACS, as described in the “Methods” (**d**). Following GAT107 treatment, a significant decrease in the expression of CD11b (macrophages), CD11c (dendritic cells), and CCR5-positive cells was observed, but there was no significant change in the number of CD4-, CD8-, and MHC class II-expressing cells (*n* = 3) (*p < 0.05, **p < 0.001)

To assess the influence of GAT107 on CNS inflammation, we treated MOG-induced EAE mice from the day of induction with GAT107 or placebo. On day 9 after treatment initiation, the mice were sacrificed. Pooled LNCs or splenocytes from both groups were re-stimulated ex vivo with the encephalitogenic MOG_35–55_ peptide (Fig. 3a), with the mitogen ConA, LPS, or anti-CD3 antibody (Fig. 3b), lymphocyte proliferation and cytokine secretion were assessed (Fig. 3a–c). LNC proliferation in GAT107-treated mice was reduced by 27% on re-stimulation with MOG_35–55_ (Fig. 3a). Similarly, in presence of the anti-CD3 antibody, ConA, and LPS, LNC proliferation was reduced by 55%, 49%, and 41% respectively (Fig. 3b). The reduction was accompanied by 94%, 80%, and 60% decrease in the secretion of pro-inflammatory cytokines IL-17, INFγ, and IL-6 respectively (Fig. 3c). In addition, a 2.8-fold increase in the secretion of the anti-inflammatory cytokine IL-10 was also observed (Fig. 3c).

### Effect of GAT107 on the expression of immune cells

Next, we tested the effects of GAT107 on immune cell populations. The phenotype of splenocytes was characterized by FACS analysis on post-immunization day 9. Interestingly, there was a significant decrease in the expression of CD11b- (macrophages), CD11c- (dendritic cells), and CCR5-positive cells (by 37%, 31%, and 45%, respectively) in the GAT107-treated mice. However, there was no significant difference in the number of CD4-, CD8-, and MHC class II-positive cells between the placebo-treated and GAT107-treated mice (Fig. 3d).

**Fig. 4:**
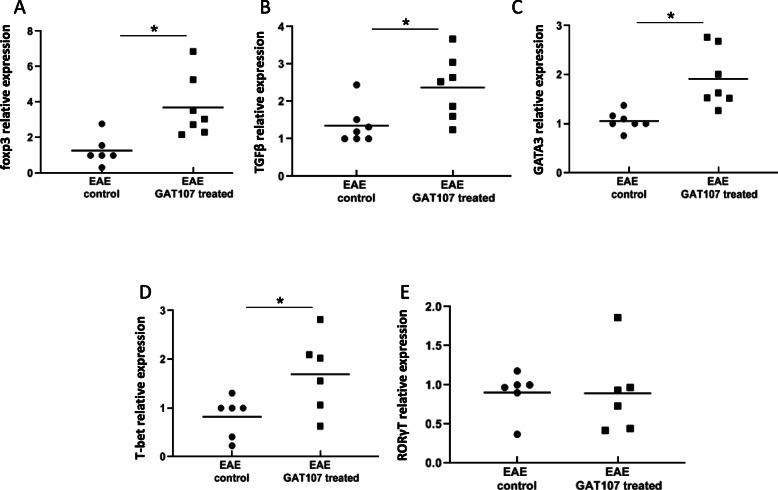
mRNA expression levels of foxp3, TGFβ, GATA3, T-bet, and RORγt following GAT107 treatment. Mice were treated with GAT107 (10 mg/day, i.p.) for 8 days from the day of EAE induction. One day later, pooled lymphocytes were obtained and activated with MOG_35–55_ as described in the “Methods”. mRNA levels of foxp3, TGFβ, GATA3, T-bet, and RORγt were assessed by RT-PCR. The results are expressed as mean relative quantification value ± SE of three separate experiments (*n* = 6–7 in each group) (*p < 0.05)

To further examine the in vivo effects of GAT107 on immune cell differentiation and proliferation, we examined the expression of several transcription factors (TFs) and TGFβ, which can serve as indicators for Th1, Th2, and Th17 as well as Treg maturation. As shown in Fig. 4, GAT107 resulted in a significant increase in the levels of T-bet (a TF for Th1), by 2-fold, and GATA-3 (a TF for Th2), by 1.8-fold. Similarly, it also resulted in an increase in IL-10. However, the expression of ROR-γt, a TF of Th17, did not change following GAT107 treatment, although a significant reduction was observed in IL-17 secretion. In addition, we found an increase in the expression of foxp3 and TGFβ, the TFs associated with regulatory T cells, by 2.9-fold and 1.7-fold, respectively.

### Effect of GAT107 on serum anti-MOG_35–55_ antibodies and B cell markers

**Fig. 5:**
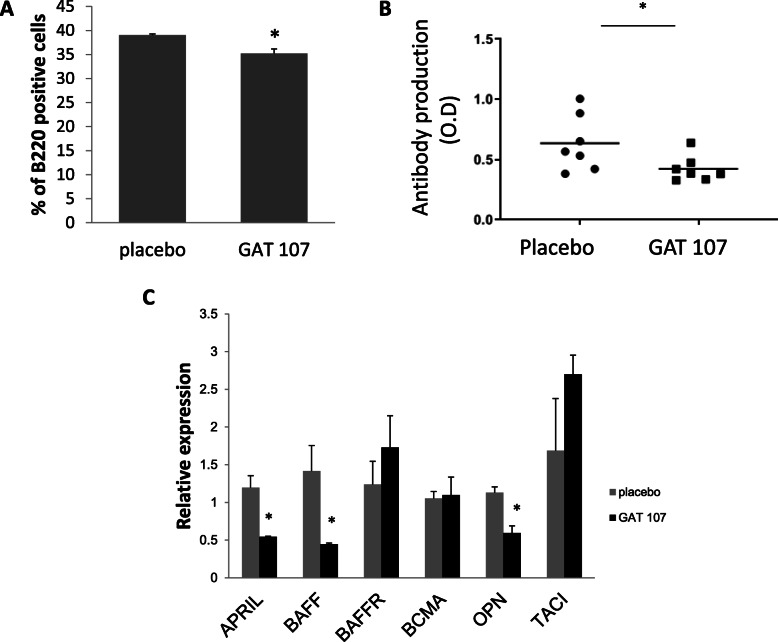
Level of B cell markers following GAT107 treatment. **a** Reduction in B200-positive cells following GAT107 treatment (*n* = 3) (*p < 0.05). **b** Reduction in anti-MOG_35–55_ antibodies following GAT107 treatment. The serum antibody levels were detected with ELISA, as described in the “Methods”. Results are expressed as the mean of two separate experiments ± SE (*n* = 7) (*p < 0.05). **c** mRNA levels of APRIL, BAFF, BAFF-R, BCMA, CXCL13, OPN, and TACI were detected by RT-PCR at day 9 after EAE induction and following ex vivo reactivation with the MOG peptide. The results expressed as mean relative quantification value ± SE of two separate experiments (*n* = 5, *p < 0.05)

Recently, the significant role of B cells in MS has come under the limelight. Therefore, we investigated the effect of GAT107 on anti-MOG antibodies and B cell markers in the EAE mice. B220, a B cell marker that represents a subset of mouse CD45 isoforms, is predominantly expressed on all B lymphocytes, including pro-mature and activated B cells, and was used as a marker of B cells. GAT107-treated mice showed a significant (9.7%) decrease in B220 expression at post-immunization day 9 (Fig. 5a). Similar results were also found at post-immunization day 30 (data not shown). In addition, the level of serum anti-MOG_35–55_ antibodies during the chronic phase of the disease (post-immunization day 30) showed a 33% reduction following treatment (Fig. 5b). This finding is consistent with the reduction in B cells, which play a role in antibody production.

To further examine the effects of GAT107 on the regulation of antibody production and B cell survival, we analyzed the expression of members of the BAFF family. Apart from BAFF, the family includes three receptors for BAFF: BAFF-receptor (BAFF-R), B cell maturation antigen (BCMA), and transmembrane activator and calcium modulator and cyclophilin ligand interactor (TACI). Two of these three receptors share the ligand APRIL (a proliferation-inducing ligand). As shown in Fig. 5c, the expression of BAFF-R, BCMA, and TACI did not significantly change in GAT107-treated mice; however, the expression of APRIL and BAFF was notably reduced by 54% and 68%, respectively. The reduction in APRIL and BAFF is consistent with the lower level of MOG_35–55_ antibodies produced and the reduction in the number of B cells and, thus, represents a less severe manifestation of the disease in the GAT107-treated mice.

Subsequently, we investigated the expression of osteopontin (OPN), which is produced by a variety of cell types, including B and T cells, macrophages, neutrophils, dendritic cells, bone cells (osteoblasts and osteocytes), and neurons [33]. As expected in less severe disease, OPN expression was significantly reduced (by 47%) in the GAT107-treated mice (Fig. 5c).

### Direct activation of α7 nicotinic AChRs by GAT107

**Fig. 6:**
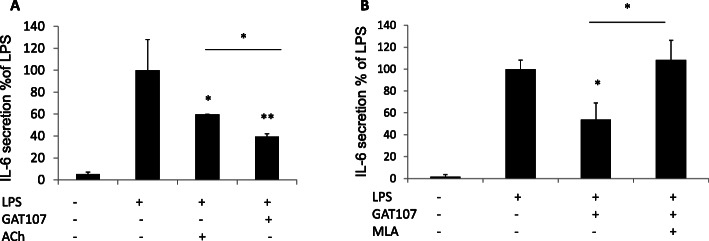
Suppressive effect of GAT107 on inflammatory cytokine release from RAW267.4 cells. IL-6 production was measured in RAW267.4 cells stimulated with LPS (25 μg/ml) with and without α7 nAChR-targeting drugs. **a** Effects of ACh (100 μM) and GAT107 (10 μM) on LPS-dependent IL-6 release. **b** MLA (10 nM) antagonizes the effects of GAT107 (10 μM) on LPS-dependent IL-6 release (*n* = 4 biological replicates, two technical replicates each) (*p < 0.05, **p < 0.01)

To examine whether GAT107 directly affects the immune cholinergic system, we examined its effect on RAW264.7, a macrophages cell line, and PBMCs from healthy donors (HDs) and MS patients. RAW264.7 cells were stimulated by LPS and incubated with agonists and antagonists of α7 nAChR (as described in the “Methods”), and the effects on IL-6 secretion were examined. As shown in Fig. 6, following LPS stimulation, ACh resulted in a 40% decrease in IL-6 secretion and GAT107 resulted in a 46% decrease in IL-6 secretion (Fig. 6a). Treatment of the cells with the α7 nAChR antagonist MLA, along with GAT107 and LPS, eliminated the anti-inflammatory effects of GAT107 (Fig. 6b). These results demonstrate that GAT107 affects cytokine release via α7 nAChR activation.

**Fig. 7:**
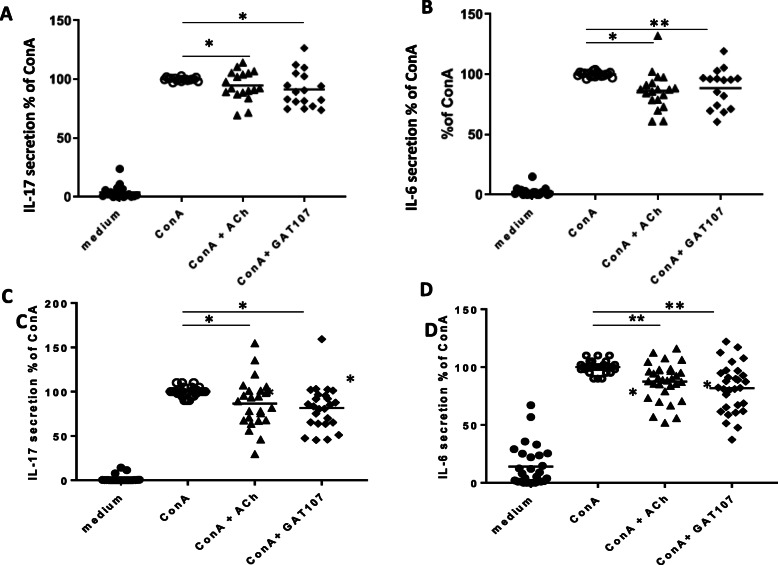
Suppressive effect of GAT107 on inflammatory cytokine release from PBMCs from HDs and MS patients. PBMCs were purified from samples from human donors, including healthy donors (**a**, **b**) and MS patients (**c**, **d**), and stimulated with ConA (5 μg/ml) with and without ACh (100 μM) or GAT107 (10 μM) for 24 h. (**a** + **c**) IL-17 and (**b** + **d**) IL-6 secretion were detected by ELISA (*n* = 17–19) (*p < 0.05)

PBMCs from HDs and MS patients were activated with ConA and incubated with GAT107 or ACh (as described in the “Methods”), and their effects on the secretion of the pro-inflammatory cytokines IL-6 and IL-17 were examined. As shown in Fig. 7, GAT107 caused a significant reduction in the secretion of both IL-6 and IL-17: IL-17 secretion was decreased by 11% in HDs and 18% in MS patients, and a similar decrease was observed after treatment with ACh (7% in HDs and 13% in MS patients). Additionally, GAT107 resulted in a 12% (HDs) and 18% (MS patients) decrease in IL-6 secretion, and ACh resulted in a 14% (HDs) and 12% (MS patients) decrease in IL-6 secretion. Treatment of GAT107- and ConA-treated cells with MLA reversed the effect of GAT107 on cytokine production (data not shown). We noticed that the effects of the cholinergic agonist on cytokine release in MS patients (Fig. 7c, d) showed higher variability than its effects in HDs (Fig. 7a, b).

## Discussion

The present study further supports the prominent involvement of the immune cholinergic system in EAE through the suppressive effects of GAT107, a dual allosteric agonist and positive allosteric modulator of α7 nAChR, on the clinical symptoms and pathological features of EAE.

In the present study, we showed that on GAT107 treatment of EAE mice, the disease severity was significantly reduced by 78% and the average time to disease onset was significantly delayed. Our results are in agreement with previous results which showed that similar cholinergic stimulants, such as rivastigmine (ACh esterase inhibitor) [7] and EN101 (ACh esterase anti-sense molecule), activated the anti-inflammatory system and, subsequently, improved EAE symptoms [6, 7]. Additionally, in this study, the neuropathological findings in the mice were consistent with the clinical severity scores and indicated that treatment with GAT107 resulted in a significant reduction in CNS neuroinflammation.

In the present study, the ameliorative effects of GAT107 were found to last for many days after treatment termination. Thus, GAT107 is likely to affect an initial stage in the cascade of events leading to CNS damage. Specifically, GAT107 may inhibit the proliferation of encephalitogenic T cells. As MS and EAE are autoimmune inflammatory diseases in which cytokines are extensively involved, accordingly, in this study, we found that Th1 and Th17 cytokines, which play a role in the induction of CNS inflammation and demyelination and in the pathogenesis of EAE and MS [1, 2, 5], were markedly reduced by GAT107 treatment. In addition, the secretion of the anti-inflammatory cytokine IL-10 was increased. Thus, our findings support the notion that GAT107 acts on encephalitogenic cells in EAE and may, therefore, have a similar effect in other diseases that involve inflammation of the CNS.

In the present study, GAT107 treatment resulted in an increase in the level of GATA-3, which is involved in Th_2_ transcription. Unexpectedly, the treatment resulted in an increase in the transcription of T-bet, while Th1 cytokine secretion was reduced. Similarly, the Th17-related TF ROR-γT was unaffected, despite the decrease in the production of Th17-related cytokines. Accordingly, a previous study from our laboratory also showed that treatment with nicotine did not affect ROR-γT expression but reduced the secretion of Th17-related cytokines [5]. Based on these findings, it is possible that GAT107 treatment influenced effector Th17 and Th1 cells but not their differentiation/maturation.

Upon studying the effects of GAT107 on specific cell types, we found a reduction in the number of macrophages (CD11b+ cells and CD11c+ cells). Macrophage activation results in the release of a range of pro-inflammatory cytokines and chemokines that contribute significantly to the expansion of the inflammatory response and associated tissue damage [34, 35].

In our study, we found a reduction in CCR5 expression following GAT107 treatment. This finding is in agreement with a previously reported study that CCR5 is involved in immune cell migration and cytokine release in the CNS [3]. Moreover, Gu et al. [36] reported that CCR5^−/−^ mice develop less severe EAE than WT mice. Additionally, they reported that patients with MS exhibit a higher percentage of circulating CCR5+ cells than control patients, and an increase in the number of these cells is associated with disease severity. These findings indicate that GAT107 action involves inhibition of T cell response, also via decrease number of CCR5+ cells. However, these findings do not imply whether the inhibition results from the reduction in the exit of those cells from the spleen or whether the treatment affect the survival and proliferation of those cells.

Tregs play a pivotal role in EAE and autoimmunity [31]. In this study, GAT107 resulted in a significant increase in the expression of foxp3 mRNA, which is a Treg marker. This finding was accompanied with an increase in the expression of TGFβ, which also is associated with Treg maturation.

However, we have not tested the expression of these markers in the CNS, nor the amount of T regulatory cells in the CNS. These experiments are planned to be performed in the future to further substantiate the effects of GAT107, and whether it is due to exit of these cells from spleen or whether the treatment affects the survival and proliferation of these cells.

Recent study in a mouse pulmonary infection with *Pseudomonas aeruginosa* model show that activation of α7 nAChRs with GAT107, decreased the bacterial burden, and improved macrophage functions [37]. Thus, we can assume that although that GAT107 has suppressive effects on myeloid populations in the spleen, GAT107 administration is not likely to affect susceptibility to other infectious**.**

Even though there is much evidence that EAE progression (or its initiation) is dependent on the activity of T cells, there is also accumulating evidence for the contribution of B cells, plasma cells, and their secreted products in the pathogenesis of EAE [38, 39]. In addition, the pathology of MS suggests the involvement of B cells in the disease course: Areas of active demyelination point to the direct interaction of myelin-specific antibodies with myelin and macrophages [40]. These findings indicate that antibody involvement is one of the pathological pathways responsible for demyelination. In the present study, the levels of anti-MOG antibodies were reduced following GAT107 treatment. This may, therefore, be one of the mechanisms involved in the reduction of axonal damage and, therefore, in the amelioration of EAE. This reduction was accompanied with a decrease in the expression of the B cell marker B220. Therefore, the level of BAFF, APRIL, and OPN, which are associated with B cell survival and function, was examined in this study. BAFF is a potent survival factor for B cells and plays an essential role in the preservation and maturation of peripheral B cells [41, 42]. BAFF expression was decreased after GAT107 treatment in this study, and this may also explain the decrease in the percentage of B cells and the lower levels of anti-MOG_35–55_ antibodies. Thangarajh et al., reported that MS patients have a higher number of APRIL-positive cells in their brain than HDs [43]. Moreover, according to Agah et al., a higher OPN level in the CSF of MS patients is associated with more active and more severe disease [44]. These findings are in accordance with the reduction in APRIL and OPN expression observed in this study and may be correlated with reduced EAE severity. Overall, they confirm the role of B cells in the anti-inflammatory mechanisms effected by GAT107 in EAE.

In the present study, in vitro direct interaction of the α7 agonist with immune cells (murine macrophage RAW264.7 cells and PBMCs from human donors) resulted in a significant decrease in the pro-inflammatory cytokines IL-6 and IL-17 (in PBMCs), and ACh directly inhibited the production of these cytokines. Additionally, the specific α7 antagonist MLA reversed the anti-inflammatory effects of GAT107 and ACh. In agreement with our findings, it has been shown that treatment with GAT107, which is specific to the α7 nicotinic receptor, activates the anti-inflammatory pathway in immune cells [8, 9, 35]. These results are also consistent with the findings of Bagdas et al., who showed that the anti-inflammatory effects of cholinergic agonists are mediated by α7 nAChR in another animal model, inflammatory and neuropathic pain [16]. Altogether, these findings confirm that GAT107 promotes the anti-inflammatory pathway via a direct effect on α7 nAChRs.

Our previous studies as well as the works of others [5, 11, 32, 45, 46] show that α7 nAChRs play an important role in the modulation of inflammatory processes via the cholinergic anti-inflammatory pathway. This pathway has been implicated in the neuroinflammation in MS [11, 45], and recently, we reported [32] that following immunological activation, α7 nAChR expression is higher in MS patients than in healthy donors. Our current data further support the ability of α7 nAChRs to respond to cholinergic agents both in MS and in healthy donors.

## Conclusion

The results presented in our study highlight the widespread immunomodulatory effects of the cholinergic anti-inflammatory pathway and the ability of the α7 nAChR-specific ago-PAM GAT107 to affect immunological processes. GAT107 treatment ameliorated EAE and modulated the cytokine pattern to an anti-inflammatory one. Moreover, GAT107 treatment affected multiple immune cell types, namely, T cells, B cells, and macrophages. Thus, α7 nAChR activation by this allosteric molecule has persistent widespread therapeutic effect on the immune response and, therefore, may have potential for the therapeutic management of inflammatory autoimmune diseases.

## Data Availability

Data will be given upon request from the co-responding authors.

## Abbreviations

BBB: Blood-brain barrier
CFA: Complete Freund's adjuvant
CNS: Central nervous system
EAE: Experimental autoimmune encephalomyelitis
FCS: Fetal calf serum
INF: Interferon
IL: Interleukin
LNC: Lymph node cell
MOG: Myelin oligodendrocyte glycoprotein
MS: Multiple sclerosis
nAChR: Nicotinic acetylcholine receptor
ago-PAM: Allosteric agonist and positive allosteric modulator
PBMCs: Peripheral blood mononuclear cells
BCMA: B cell maturation antigen
TACI: Transmembrane activator and calcium modulator and cyclophilin ligand interactor
APRIL: A proliferation-inducing ligand
BAFF: B cell activating factor
BAFF-R: B cell activating factor receptor
OPN: Osteopontin
Ach: Acetylcholine
MLA: Methyllycaconitine
